# circDYRK1A tethers biological behaviors of gastric carcinoma using novel bioinformatics analysis and experimental validations

**DOI:** 10.1038/s41598-023-33861-1

**Published:** 2023-05-22

**Authors:** Zirui Zhu, Huiwen Lu, Xu Zhao, Yimeng Sun, Junqiao Yao, Chi Xue, Baojun Huang

**Affiliations:** 1 Department of Surgical Oncology and General Surgery, The First Hospital of China Medical University, Key Laboratory of Precision Diagnosis and Treatment of Gastrointestinal Tumors (China Medical University), Ministry of Education, No.155 Nanjing North Street, Heping District, Shenyang, 110001 China; 2 School of Medicine, Xiamen University, Xiamen, 361000 China

**Keywords:** Cancer, Cell biology, Computational biology and bioinformatics, Molecular biology, Biomarkers, Oncology

## Abstract

Gastric cancer has been one of the wide public health burdens with its high morbidity and mortality over several decades. As the unconventional modules among RNA families, circular RNAs present their blazing biological effects during gastric carcinogenesis. Though diverse hypothetical mechanisms were reported, further tests were necessitated for authentication. Herein, this study pinpointed a representative circDYRK1A which screened from vast amounts of public data sets using surprisingly novel bioinformatics approaches together with validations from the in vitro findings and then concluded that circDYRK1A tethered the biological behavior and swayed the clinicopathological features with gastric cancer patients thus providing an in-depth awareness for gastric carcinoma.

## Introduction

As one of the most common malignancies worldwide, gastric carcinoma (GC) remains the third leading cause of cancer-associated deaths in less developed countries in 2018^1^. While already substantial efforts have been made in individual therapy and management for GC patients, the clinical outcomes and prognosis of which are still unfavorable, with the 5-year overall survival (OS) rate less than 30%^2^. The high mortality is very due primarily to its late diagnosis when extensive tissue invasion and distant organ even peritoneal metastasis has occurred. It is therefore urgent to identify new potential biomarkers and therapeutic targeted candidates for earlier monitorization and diagnosis thus ameliorating the clinical outcomes of GC patients.

International Human Genome Sequencing Consortium has revealed that around 2% of the human genome encodes for protein-coding genes, while the rest majority of the genomic landscape consists of non-coding elements like long non-coding RNAs (lncRNAs), microRNAs (miRNAs), or circular RNAs (circRNAs)^3^. CircRNA, a lately recognized group of particular non-coding RNA molecules characterized by covalent and closed loop structures, represents a number of striking futures and functions compared to its counterparts. Since the structure absence of a 5′-cap or 3′-polyadenylated tail, circRNAs with the capability of resisting the degradation by exonuclease RNase R exhibit much more structural stability in certain organs and tissues than linear RNAs^4–6^. Specifically, a preponderance of evidence has been revealed that circRNAs exert a considerable influence on the course of oncogenes and cancer development attributing to their diverse biological actions during proliferation, invasiveness, migration, and metastasis of malignant cells^7–9^. Relevant researches involved in circRNAs have been piecemeal carried out for over 50 years from their inceptive recognition in 1971, nevertheless, circRNAs remain widely regarded as the accidental splicing intermediate or by-products of splicing errors, and their molecular and mechanistic events responsible for GC are still largely inexplicit^10^. Given a swift pace in high-throughput RNA sequencing (RNA-Seq) technology attached to the bioinformatics algorithms over the last few decades, circRNAs, to a great extent, have been elucidated and some of the rudimentary functions also uncovered, such as sponge miRNAs as miRNA response elements (MREs)^11^, alternative splicing to regulate the gene expression through unproductive splicing, modulating the transcriptional level in their parental genes^12^, stably combining with AGO proteins and Pol II, and there is no evidence to exclude other RNA-binding proteins (RBPs) as well^13^. In addition, another fact also worth mentioning is that a small fraction of them has the ability to be translated into protein modules^14^. As it stands, serving as miRNA-sponges that sequester miRNA molecules remains one of the best-characterized functions of circRNAs, also dubbed competitive endogenous RNA regulation mechanism (ceRNA)^15–17^. As one example, circRNA_100269 regulates the capability of proliferation in GC cells by sponging miR-630 and thus as a biomarker used to predict cancer recurrence simultaneously^18^. An additional study found that the overexpression of circCUL2 impeded tumorigenicity and malignant transformation of GC and may act as a tumor negative regulator through miR-142-3p/ROCK2-mediated activation of autophagy, which could be a pivotal underlying mechanism of GC^19^. Another research showed that circLMTK2 facilitated tumor proliferation, division, and metastasis in gastric cancer through sponging miR-150-5p, in the meantime, a highly-expressed level of circLMTK2 was correlated with a higher number of lymph nodes with metastases, poor TNM (Tumor Node Metastasis) classification, and displeasing prognosis in GC patients^20^.

Despite such fascinating signs of progress in circRNA modules have been made, the counts and identities of which engaged in circRNA biological regulatory interactions are still greatly ill-defined. In the present study, we focused on this representative circDYRK1A screened from vast amounts of biological data exist combined with novel bioinformatics methods. What’s more, we further verified the expression pattern and the GC-based biological roles of circDYRK1A at the level of experiments to make our bioinformatics hypothesis more convincing.

## Methods

### Data sources

The ArrayStar microarray profile matrix files acquired in the current research were analyzed and downloaded from the Gene Expression Omnibus (GEO, https://www.ncbi.nlm.nih.gov/gds/) databank which is totally available to the public. A total of 5 differentially co-expressed circRNAs were recognized in GSE89143 (GEO Series, 6 chip samples containing 3 mates of GC tissue cases and adjacent-benign controls), GSE93541 (6 chip samples containing 3 mates of GC plasma cases and non-tumor controls), and GSE100170 (10 chip samples containing 5 mates of GC tissue cases and adjacent-benign controls). The miRNA profile matrix data were retrieved from datasets GSE54397 (32 chip samples containing 16 mates of GC tissue cases and adjacent-benign controls), GSE78091 (6 chip samples containing 3 mates of GC tissue cases and adjacent-benign controls), and GSE93415 (40 chip samples containing 20 mates of GC tissue cases and adjacent-benign controls). The mRNA profile matrix data were withdrawn from GSE54129 (132 chip samples containing 111 GC tissue sets and 21 non-tumor controls). The whole expression profiles within these GSE datasets were subject to standardized signal intensity.

### Recognition of differential RNAs expression

The microarray profile sets downloaded containing platform records together with series matrix files were both conducted with the help of the latest R language (as well as its relevant annotation packages) and Perl software. CircRNA ID annotations were further translated into the globally acknowledged circRNA normalized jargons (circRNA symbols). The recognition of differentially expressed circRNAs (DEcircRNAs) firstly proceeded under the aegis of Bioconductor Limma package with an inclusion criterion P-value < 0.05, and later we took an intersection of the three processed GSE series, the robust rank aggregation algorithm was further carried out to rank the DEcircRNAs, and co-expressed circRNAs in the same direction (i.e., co-up-regulated or co-down-regulated) were considered as our finally significant DEcircRNAs^21^. In addition, differentially expressed miRNAs (DEmiRNAs) in datasets GSE54397, GSE78091 and GSE93415 were distinguished via GEO2R, an online analytic program in GEO website, and the False Discovery Rate (FDR) values < 0.05 along with the absolute values of log2 Fold Change (|Log2FC|) > 0.05 were reasonably assumed to be statistically significant due to the |Log2FC| values amid most miRNAs were less than 1. What's more, differentially expressed mRNAs (DEmRNAs) in dataset GSE54129 were additionally screened with inclusive criteria that FDR values < 0.05 plus |Log2FC| values > 1 using GEO2R tools.

### Prediction of miRNA-sponged sites

5 significantly screened DEcircRNAs in the same co-expressed direction were integrated with the robust rank aggregation (RRA) method. Then we respectively forecasted the miRNA-sponged sites (also dubbed miRNA response elements, MREs) among the five DEcircRNAs through the combination of two internationally accepted miRNAs prediction websites, The Cancer-Specific CircRNA Disease (CSCD) (http://gb.whu.edu.cn/CSCD/) and The Circular RNA Interactome (CircInteractome) (https://circinteractome.nia.nih.gov/). Additionally, we computed the convergence portions from the results of CSCD, CircInteractome, GSE54397, GSE78091, and GSE93415, then finally, the number of overlapping data sets ≥ 3 together with at least one intersection from CSCD or CircInteractome was viewed as our inclusive criterion to obtain our ultima DEmiRNAs.

### Prediction of miRNA-targeted genes

Thanks to the prevalence of R language and Perl script, miRNA-targeted genes were sifted based upon the 3 gene predictions online tools, which contain miRTarBase (http://mirtarbase.mbc.nctu.edu.tw/), miRDB (http://www.mirdb.org/), and TargetScan (http://www.targetscan.org/)^22–24^. However, not entire findings were brought into this research, mutually shared sections among the three databases were considered as significant candidates. Similarly, we obtained the united junctions of the candidates along with the differentially expressed portions from dataset GSE54129 to acquire our ultima mRNAs.

### Visualization of ceRNA relationships

Following all the predicted differentially expressed ultima RNAs, a circRNA-based alluvial regulatory interaction was visualized according to the blend of circRNA-miRNA mates in conjunction with miRNA-mRNA mates via http://www.bioinformatics.com.cn, a free online platform for data analysis and visualization.

### Comprehensive function analysis

With the purpose of scrutinizing the potentially functional mRNAs among the ceRNA triples coupled with their potency to the molecular biology in GC, the Kyoto Encyclopedia of Genes and Genomes (KEGG) pathway was performed using R language and the top-ten most significant pathway terms of which were mapped into a bubble chart with the screening threshold of P values < 0.05^25–27^. Furthermore, a web-based site DAVID (version 6.8, https://david-d.ncifcrf.gov) for functional enrichment analysis was employed to calculate the meaningful enrichment of interrelated genes within the KEGG annotations. More importantly, a co-relevant network of the KEGG terms together with pathways-related genes was put forward by virtue of Cytoscape, and the number of genes integrated with KEGG terms no less than 4 was considered as our inclusion criterion. In addition, the STRING (https://string-db.org/) analysis tool was exerted to design a general inter-connection landscape based upon the ultima mRNAs pinpointed. The interdependent genes were then visualized using Cytoscape, and above all, the most momentous top-ten nodes (hub genes) ranked by Degree algorithm by dint of its plugin CytoHubba.

### Cell culture and transfection

Three human GC cell lines containing HGC-27, AGS, and MGC-803 together with one human gastric epithelial GES-1 cell were applied in this study from China Medical University. All these cells were respectively cultured in Dulbecco's Modified Eagle's Medium (DMEM, Gibco, USA) with 10% fetal bovine serum (FBS, Gibco, USA) concentration and then preserved in a moisturized incubator containing 5% CO_2_ concentration at 37 °C for nearly 24 h. RNAs were extracted among our GC cell lines and healthy GES-1 control, and the GC cell line with the lowest expression level of circDYRK1A was considered as the subsequent research object. The cDNA fragment was cloned into the pcDNA3.1(+) vector (Suzhou Gemma) to up-regulate circDYRK1A, and the over-expressed plasmid was constructed and then transfected into our targeted cell line using Lipofectamine 2000 Reagent (Invitrogen Company, USA) based upon the manufacturer's protocol. The relative expression values of circDYRK1A in the transfected cell lines were detected via Quantitative PCR (q-PCR) Analysis.

### Patients and tissue collection

Clinical tissue specimens of paired GC tissues and adjoining healthy counterparts (n = 80) were collected from patients who underwent radical surgical operation between October 2019 and August 2020 in the First Affiliated Hospital of China Medical University. All specimens isolated were stored quickly at − 80 °C after collecting in RNA stabilization solution (Invitrogen, by Thermo Fisher Scientific). For samples-expand, clinical and pathological features of which comprising Age, Gender, Tumor size, Differentiation, Tumor Stage, Lymphatic metastasis, and pTNM (pathological Tumor Node Metastasis) classification were accurately analyzed. None of the patients had instilled any radiotherapeutic or chemotherapeutic treatment prior to surgical procedures. All participants and their legal guardians were completed a predesigned written informed consent prior to study participation, meanwhile, ethics approval was received from the Institutional Ethical Committee of China Medical University before this research. More importantly, all methods were performed in accordance with relevant guidelines and regulations.

### RNA isolation and qRT-PCR analysis

Total RNA isolation was carried out using TRIzol reagent kit (Invitrogen, USA) and the complementary DNA (cDNA) synthesis was carried out through PrimeScript RT reagent kit (Takara, Japan) following their manufacturers’ recommendations. The quantitative real-time PCR (qRT-PCR, Roche, Switzerland) was completed via SYBR green fluorescent dye assay (Power SYBR Green, Applied Biosystems). The expression degree of circDYRK1A had standardized to glyceraldehyde 3-phosphate dehydrogenase (GAPDH) against the algorithm of 2^−∆∆CT^. The primer sequences which designed by Sangon Biotech (Shanghai, China) adapted for qRT-PCR were as follows:

CircDYRK1A-F: CAGCATTGTCAGCTCCTGGA

CircDYRK1A-R: AAGAGTCCAGCGGCAAAACT

GADPH-F: GGAGCGAGATCCCTCCAAAAT

GADPH-R: GGCTGTTGTCATACTTCTCATGG

### Cell proliferation assay

Cell Counting Kit-8 (CCK-8, Beyotime, Shanghai, China) test was operated to gauge the proliferative ability of cells following the operation steps of the manufacturer’s information. Transfected cells were inoculated into 96 well plates on the concentration of 5 × 10^4^/well (100 μL per well) for a stationary culture under recommended cell-specific ambient conditions. The assay was carried out with no less than three duplicate wells. CCK‐8 solution (10 μL) was attached at the time point of 0th, 24th, 48th, 72nd, and 96th hours with incubation environments of 37 °C and 5% CO_2_. In the end, cell viability was measured based upon the optical density (OD) value per well at a wavelength of 450 nm (OD 450) with an automatic microplate reader.

### Cell invasion assay

Transwell test was conducted to measure the invasive capacity of the preferred cell line in Transwell chambers. Matrigel Basement Membrane Matrix purchased from BD Biosciences was firstly coated into the inner layer of the Transwell chamber. After 24 h of transfection, the cells were starved for about 12 h on serum-free DMEM, and the concentration of cells was adjusted to about 1 × 10^6^/mL. In each chamber, cell suspension was placed into the upper part, and DMEM containing 10% FBS was placed into the lower part. At last, the number of metastatic cell invasions into the opposite side of the Matrigel membrane were counted against the optical microscope (× 200).

### Cell migration assay

Scratch Wound Healing Test was measured to test the cell migrative mobility. The back of the six-well plate was perpendicular to the horizontal axis, and marks were made at approximately 1.0 cm intervals. According to the concentration of 5 × 10^5^/well, cells in the logarithmic growth phase and not treated with serum within 24 h were seeded into six-well plates. When the cells reached 90% confluency, we used a pipette tip to scratch the cells along the marked line. Eventually, the scratched lines were photo-imaged and the migration rate was calculated under timepoint 0 h and 48 h.

### Statistical analysis

All statistical enumeration and measurement outcomes were subjected to Student t-test or one-way ANOVA using SPSS Statistics (version 26.0). The P-value of less than 0.05 (*P* < 0.05) was deemed to be statistically significant with *P < 0.05, **P < 0.01, ***P < 0.001 and ****P < 0.0001, and all these experiment sets were reproduced no less than thrice. Besides, all drawings were generated and visualized using Prism software (version 8.2.0).

## Results

### Determination of DEcircRNAs, DEmiRNAs, DEmRNAs

**Figure 1:**
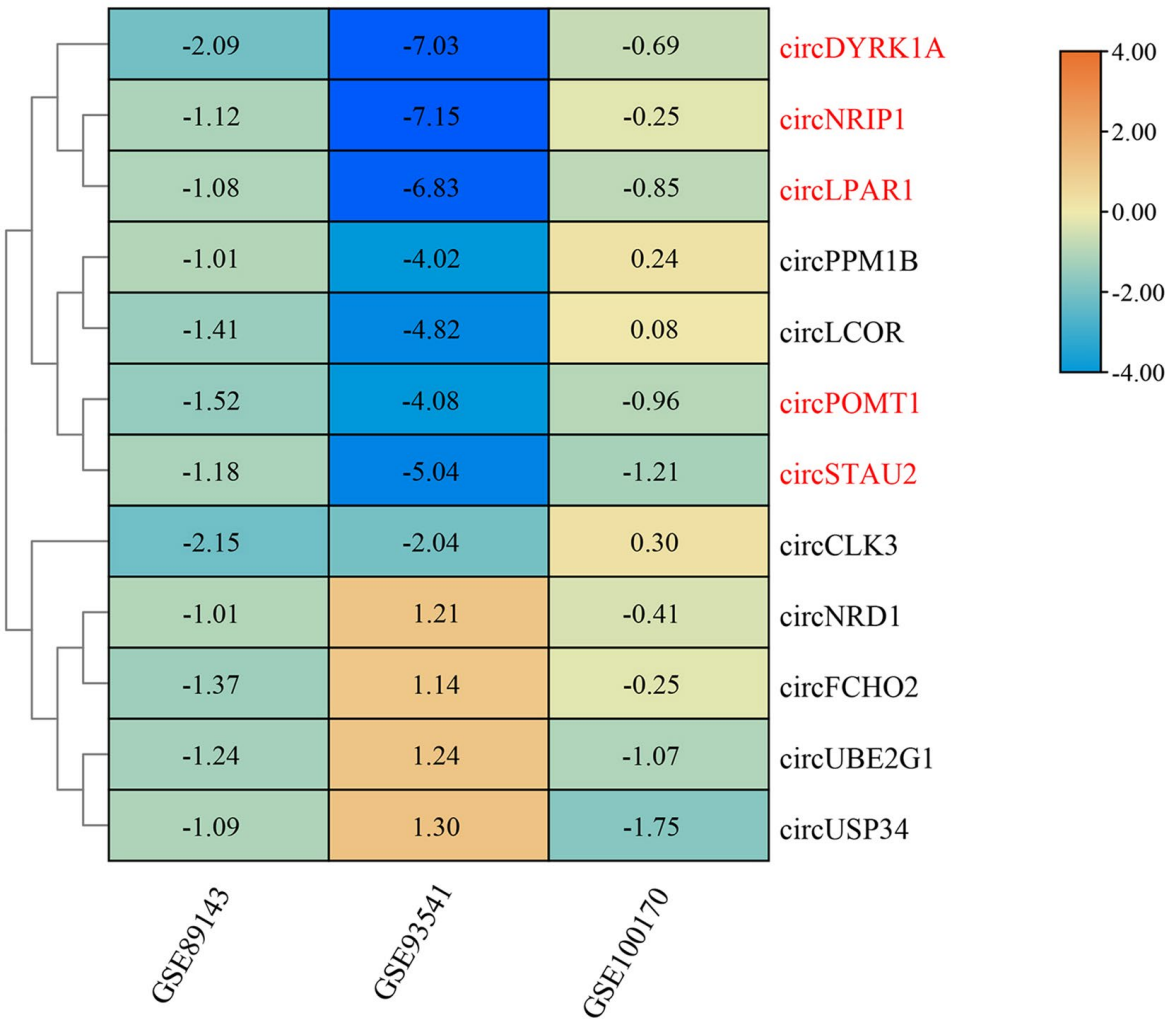
Heatmap of the five differentially co-expressed circRNAs. The X-axis represents three GEO Series (GSE), and the Y-axis represents the clustered circRNAs. The five circRNAs are highlighted in red. The numbers indicate the relative expression of circRNAs, with positive numbers indicating high expression and negative numbers indicating low expression. The lower expression of circRNA is bluer in color and smaller in number, and the higher expression of circRNA is more orange in color and larger in number.

Sets of DEcircRNAs were sifted over the three circRNAs microarray GEO series. Altogether, 53 DEcircRNAs (8 up-regulation and 45 down-regulation) were totally discerned in GSE89143, 306 DEcircRNAs (146 up-regulation and 160 down-regulation) were totally discerned in GSE93541, 1414 DEcircRNAs (537 up-regulation and 877 down-regulation) were totally discerned in GSE100170. Then the DEcircRNAs within every one of the three diverse datasets were amalgamated and ranked, and a total of 12 entrants were manifested in the top rank using the heatmap module (P values < 0.05). Eventually, those co-expressed candidates in the same direction (i.e., co-up-regulation or co-down-regulation) were viewed as our ultima DEcircRNAs, which were presented as circDYRK1A (also dubbed circDYRK1A), hsa_circ_0004771, hsa_circ_0061276, hsa_circ_0001897, and hsa_circ_0001811 (Fig. 1). Similarly, differentially expressed miRNAs sets were separated within the three miRNAs microarray GEO series. Completely, 368 DEmiRNAs (153 up-regulation and 215 down-regulation) were picked in GSE54397, 795 DEmiRNAs (643 up-regulation and 152 down-regulation) were picked in GSE78091, 629 DEmiRNAs (264 up-regulation and 365 down-regulation) were picked in GSE93415. What is more, the equivalent approach was carried out in mRNA microarray GSE54129, and a total set of 4345 DEmRNAs were distinguished, with 482 up-regulation and 3863 down-regulation.

### Determination of targeted sites

**Figure 2:**
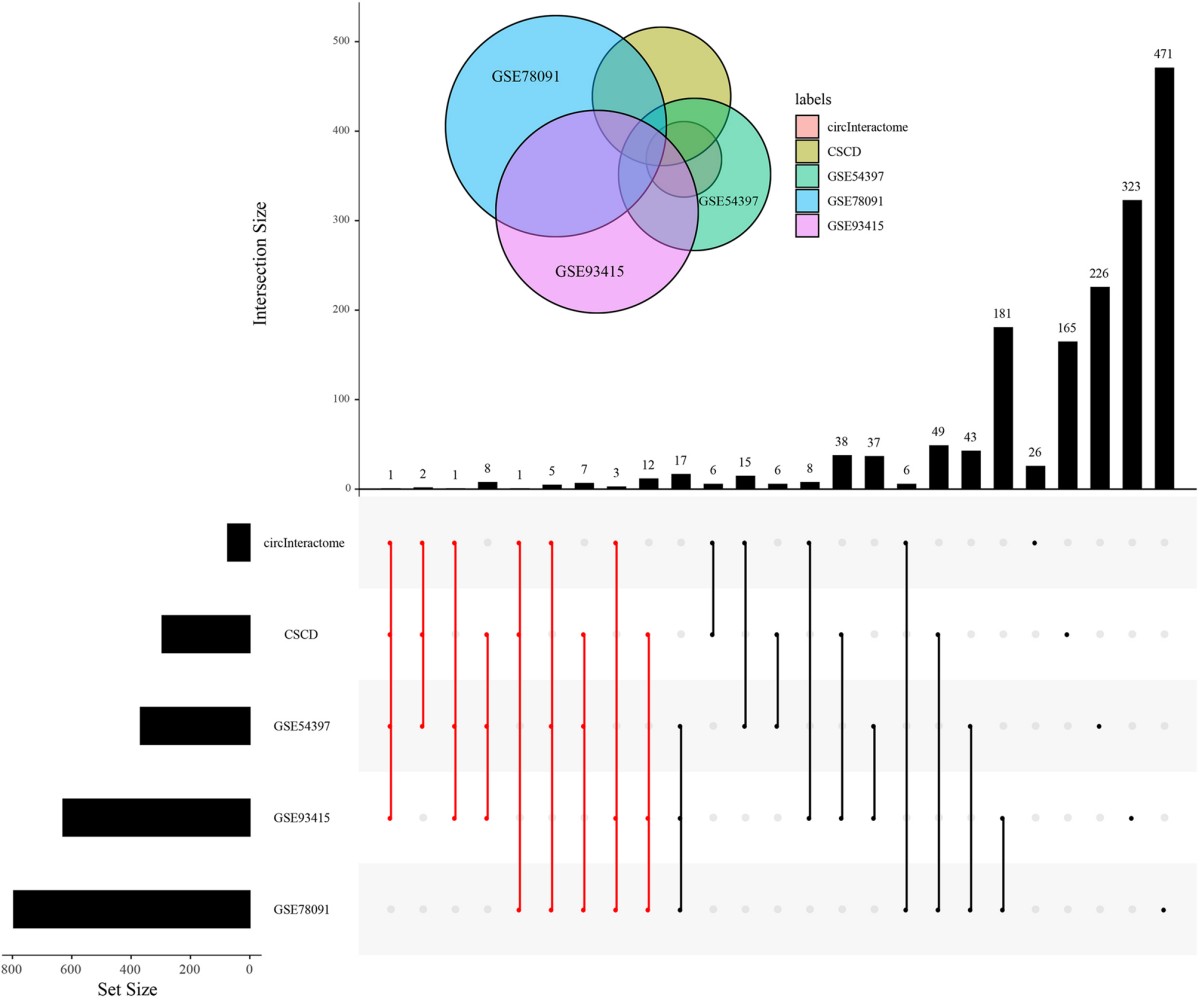
The targeted miRNAs intersection among CircInteractome, CSCD, and three GSE data. The red dots indicate our inclusive miRNA objects, the vertical lines represent the objects of the intersection, the length of the vertical black column represents the amount of incomplete intersection data included, and the Set Size is the size of the sample.

In accordance with our inclusive criterion of targeted miRNAs sites, entirely, 40 ultima DEmiRNAs were extracted and then visualized among the manifold crossing of CircInteractome, CSCD, GSE54397, GSE78091, and GSE93415 (Fig. 2). Besides, with regard to the targeted mRNAs locus prediction of ultima DEmiRNAs, 236 of which were eventually filtered via the overlapped intersection of 3 gene predictions tools together with differentially expressed portions from the dataset GSE54129.

### Visualization of ceRNA triples

**Figure 3:**
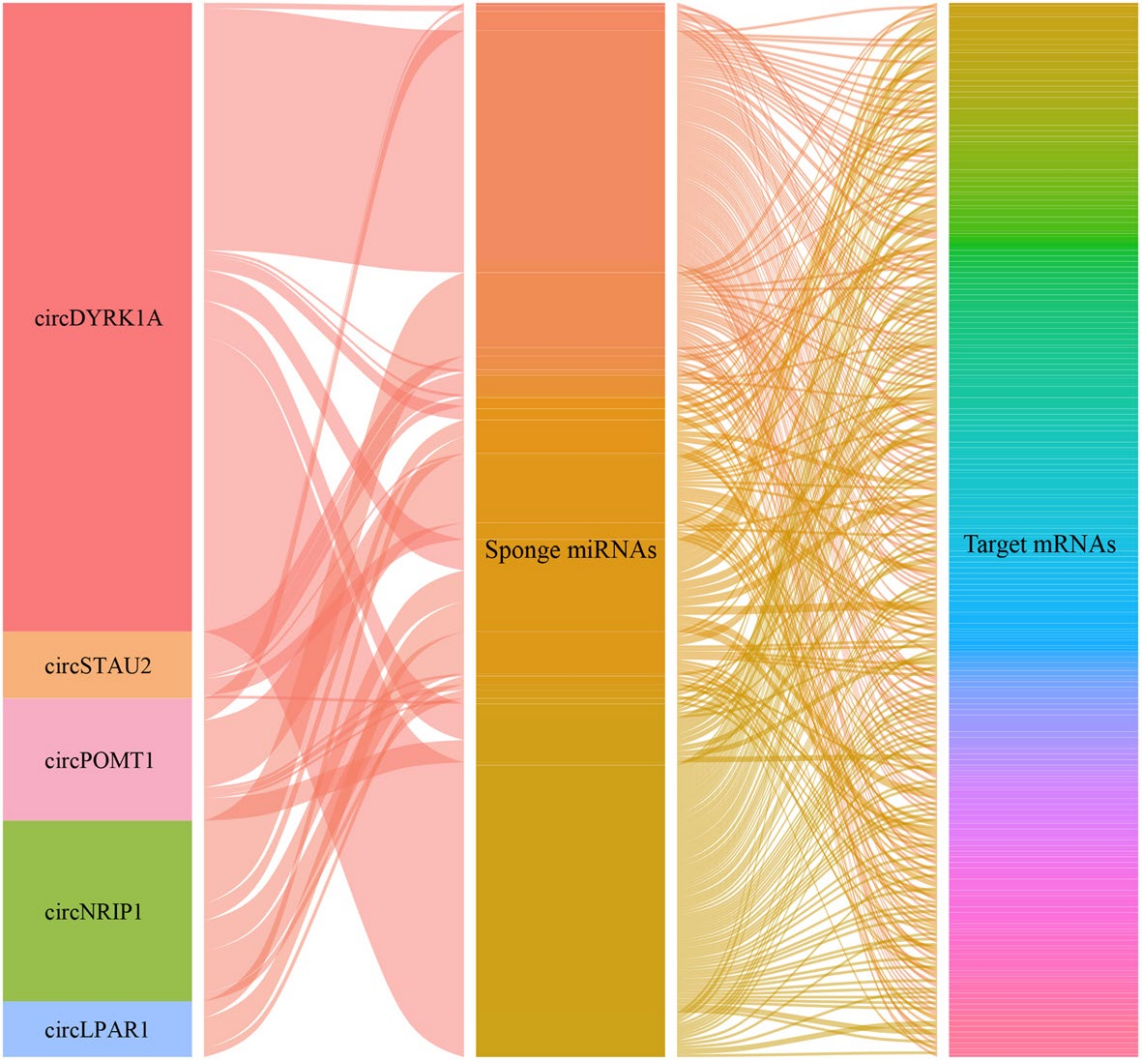
Alluvial ceRNA plot constructed among the triples of circRNAs, sponge miRNAs, and target mRNAs. 5 differentially expressed circRNAs are in the leftmost column of the figure, 40 differentially expressed miRNAs are in the middle column of the figure, and 236 differentially expressed mRNAs are in the rightmost column of the figure. Different colors represent different objects. The width of the alluvial lines represents the number of enrichments.

CeRNA regulatory network was visualized for a strong and intuitionistic understanding of the shared relationships among the triples of circRNAs, miRNAs, and mRNAs. In consideration of the former-analytical consequences, 5 congregative down-regulated ultima DEcircRNAs, 40 sponge miRNAs including 36 up-regulation and 4 down-regulation, and 236 target mRNAs including 32 up-regulation and 204 down-regulation and their all bonds were outlined by means of alluvial ceRNA network, and the width of the alluvial lines represents the number of enrichment (Fig. 3).

### KEGG-related analysis of mRNAs

**Figure 4:**
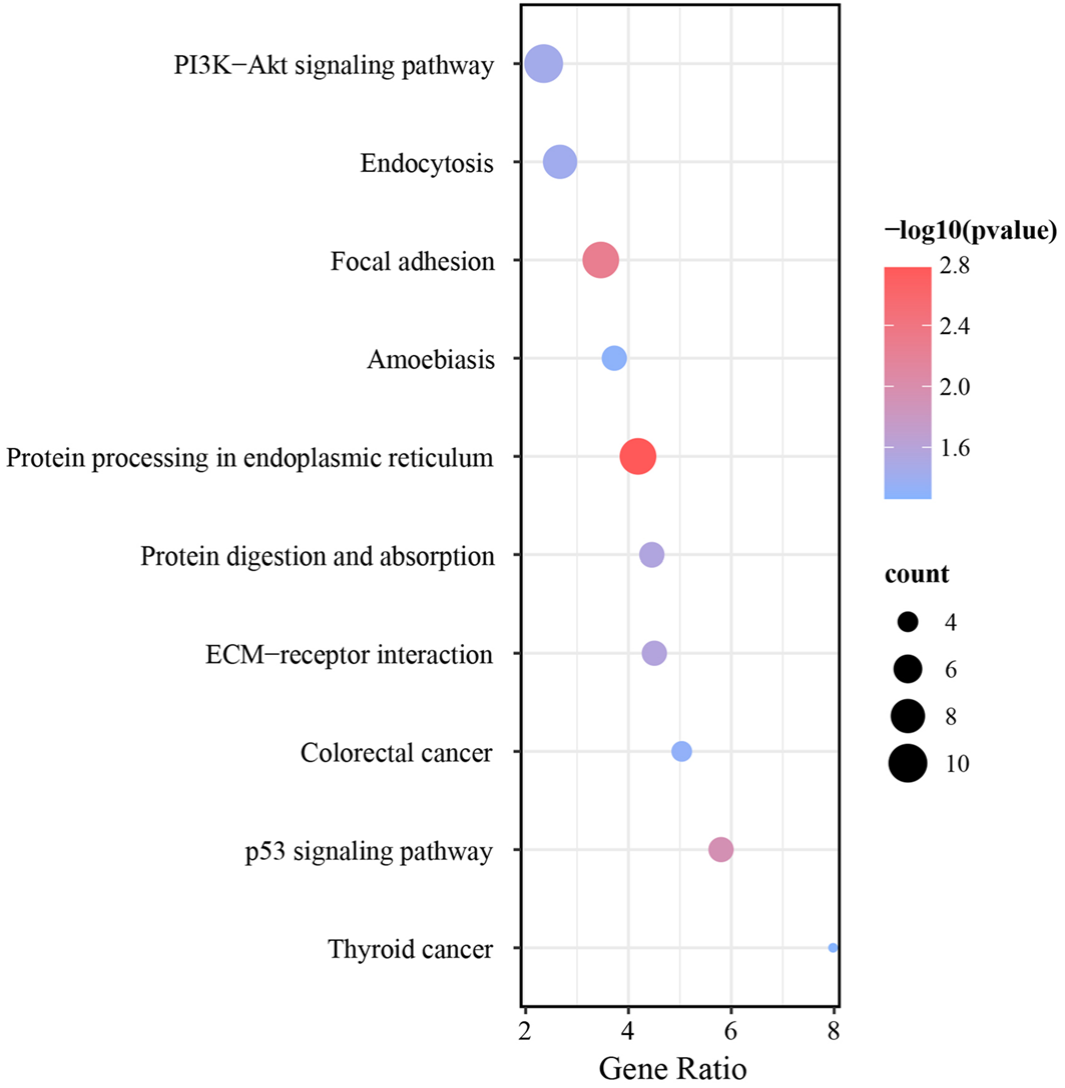
Top-ten most KEGG-enriched annotations involved in the target mRNAs. The abscissa Gene Ratio indicates the ratio of the gene enrichment number of this entry to the total gene enrichment number of the entry. The ordinate axis represents the name of the KEGG pathways. The size of the dots represents the number of genes involved (count), and the larger the dots, the greater the number of genes contained in the KEGG pathways. The color of the dots represents the degree of enrichment, which is -log10(pvalue) in the figure. The redder the dots, the higher the degree of enrichment, and the greater the importance of the pathway; the bluer the dots, the lower the degree of enrichment, and the lower the importance of the pathway.

**Figure 5:**
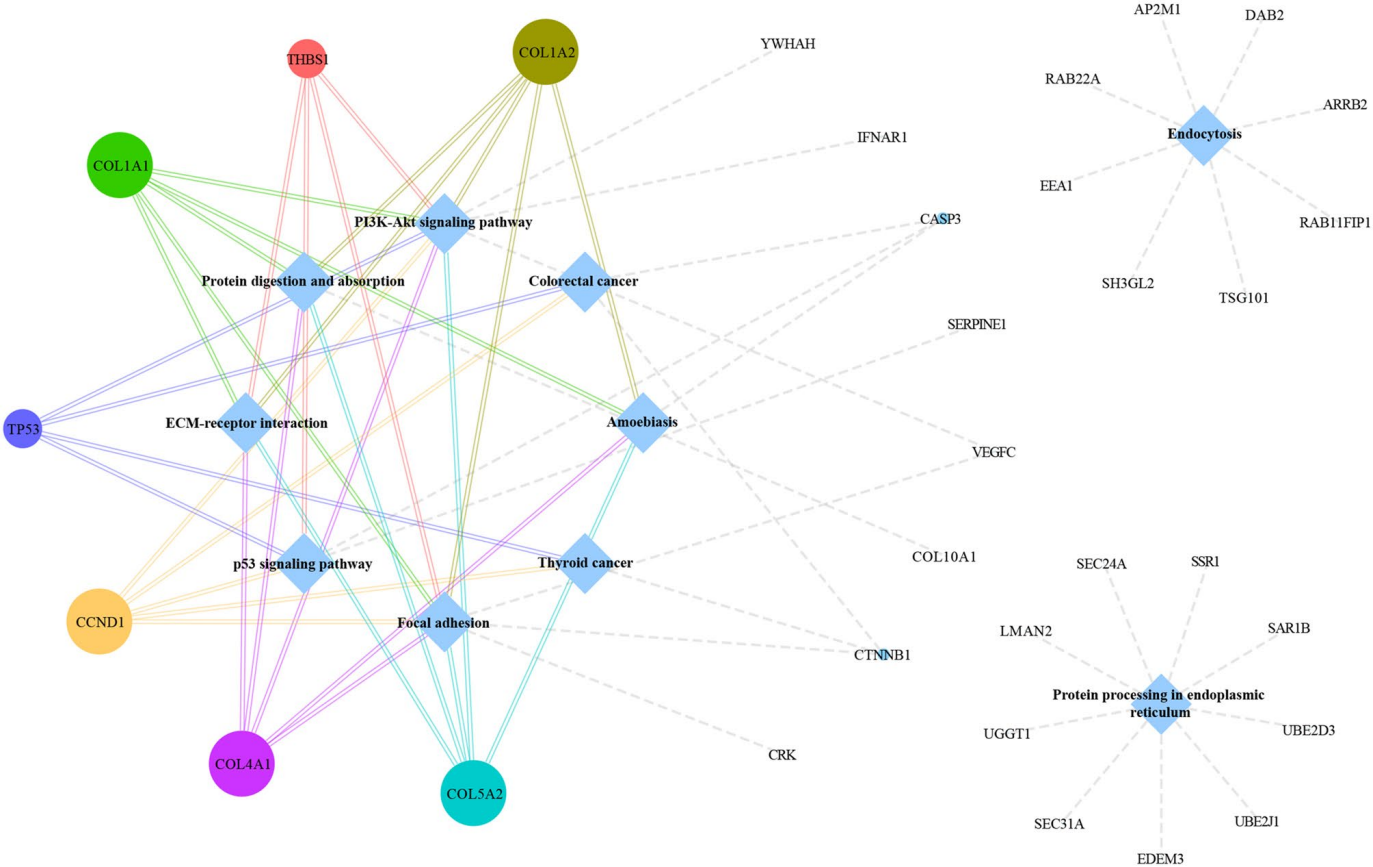
The network consisted of the most significantly KEGG-enriched pathways and their inter-related target genes. The circles represent the genes enriched in KEGG pathways, and the diamonds represent the top 10 KEGG pathways of gene enrichment items. The size of the circle reflects the number of pathways that target genes enriched, and the larger the circle, the more times the gene is enriched in the KEGG pathway.

Following, 236 target mRNAs were further exploited through KEGG-related analysis to delve into the potentially functional circRNA we were interested in. The inclusive pathways were ordered according to the normalized enrichment score (NES) calculation and the top-ten most enriched KEGG-based annotations among these modules were constructed, meanwhile, mRNAs count as well as the − log10(P-value), namely their hypothesis testing parameters, were all displayed in Fig. 4, in which the thyroid cancer and p53 signaling pathway were significantly enriched with the maximum score values. Besides, the network comprised of the top-ten KEGG terms together with pathways-related genes manifested that COL1A1, COL1A2, COL4A1, COL5A2, TP53, THBS1, and CCND1 were all substantial cross mRNAs that engaged in no less than four KEGG signal annotations (Fig. 5).

### Comprehensive interaction analysis of target mRNAs

**Figure 6:**
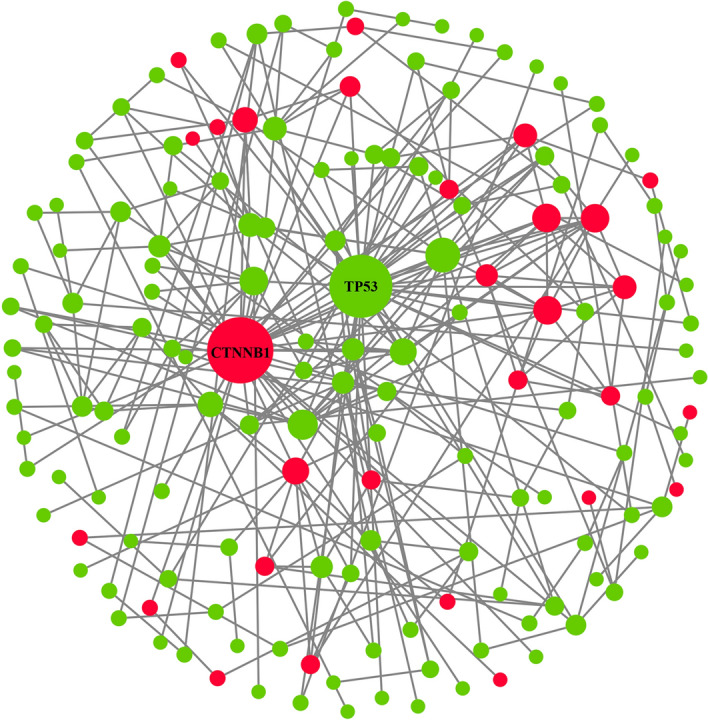
Interaction landscape of target mRNAs. Genes are represented by circular blocks, with red representing up-regulated gene expression and green representing down-regulated gene expression. The size of the circle represents the enrichment score of the gene node, and the larger the circle, the more genes interact with this gene.

**Figure 7:**
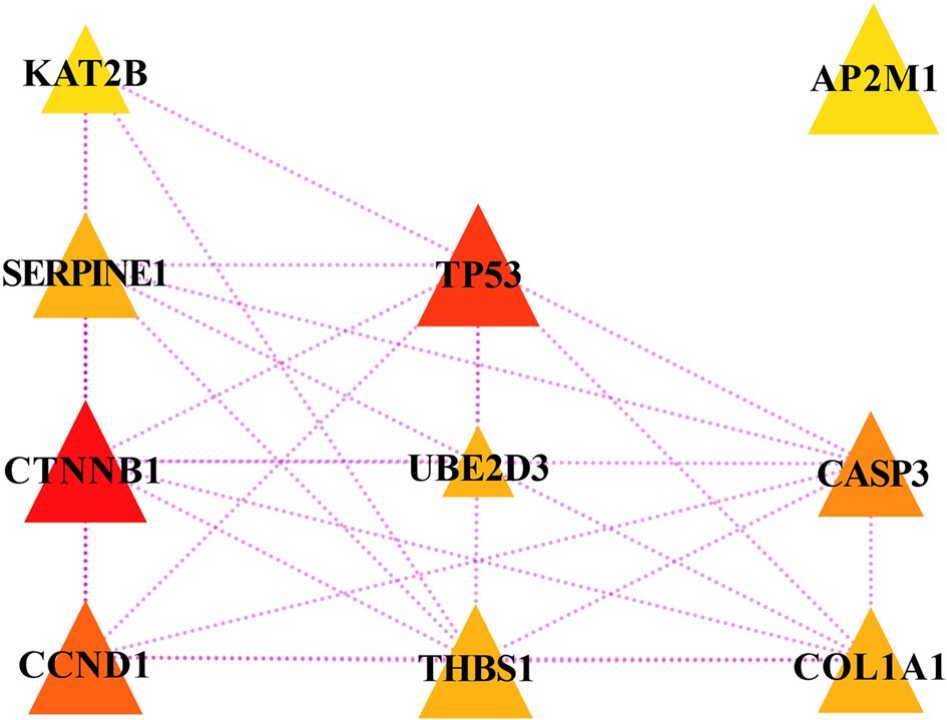
Interaction network of hub genes. The gene is represented by a triangle block. The relative size and color of the triangle represent the degree of interaction of hub gene using Degree method. The larger the gene block or the redder the color, the more genes interact with hub gene; the smaller the gene block or the more yellow the color, the fewer genes interact with hub gene.

The STRING analysis tool and Cytoscape as well as its plugin were utilized to explore the one-to-one item association along with the hub genes among 236 ultima mRNAs. The relative scale of which was calculated according to Degree algorithm in Plugin CytoHubba (Fig. 6), and the cross-talk network of the most momentous top-ten nodes (hub genes) ranked in the same way was unfolded in Fig. 7. In addition, we found that two of hub genes, TP53 and CTNNB1, were extremely inter-correlated genes that linked eight counterparts.

### Determination of aimed circRNA

We discovered that COL1A1, COL1A2, COL4A1, COL5A2, TP53, THBS1, and CCND1 were all substantial candidates in the top-ten KEGG terms together with pathways-related genes, and simultaneously, TP53 and CTNNB1 were all substantial candidates in the cross-talk network of the most momentous top-ten hub genes. By the combination of the outcomes within the two distinct approaches, we concluded that TP53 was the most substantially meaningful mRNAs according to the data and methods we provided. Ultimately, we retrospectively determined that circDYRK1A/hsa-miR-485-5p/TP53 was the final regulatory axis by combining the former alluvial ceRNA components network and selected circDYRK1A as our research subject in the present study.

### Expression of circDYRK1A in vitro cell lines and vivo tissues

**Figure 8:**
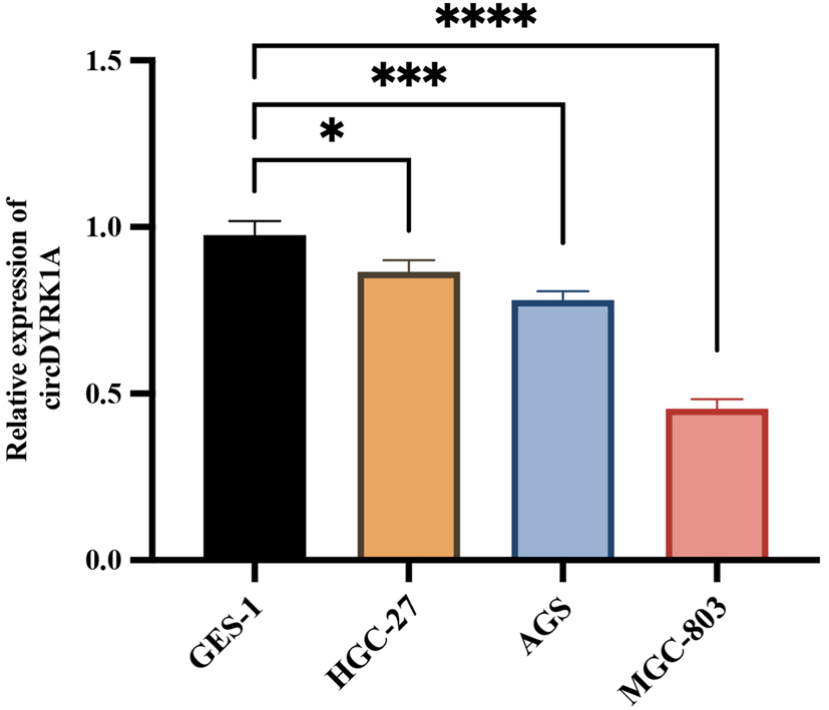
Relative expression of circDYRK1A among three GC cell lines in comparison to GES-1. Compared with GES-1, the expression of circDYRK1A was down-regulated in HGC-27 (*P* < 0.1), the expression of circDYRK1A was down-regulated in AGS (*P* < 0.001), and the expression of circDYRK1A was down-regulated in MGC-803 (*P* < 0.0001). The X-axis is the four cell lines and the Y-axis is the 2^-ΔΔCt^ value, and the higher the value, the higher the relative expression of circDYRK1A.

**Figure 9:**
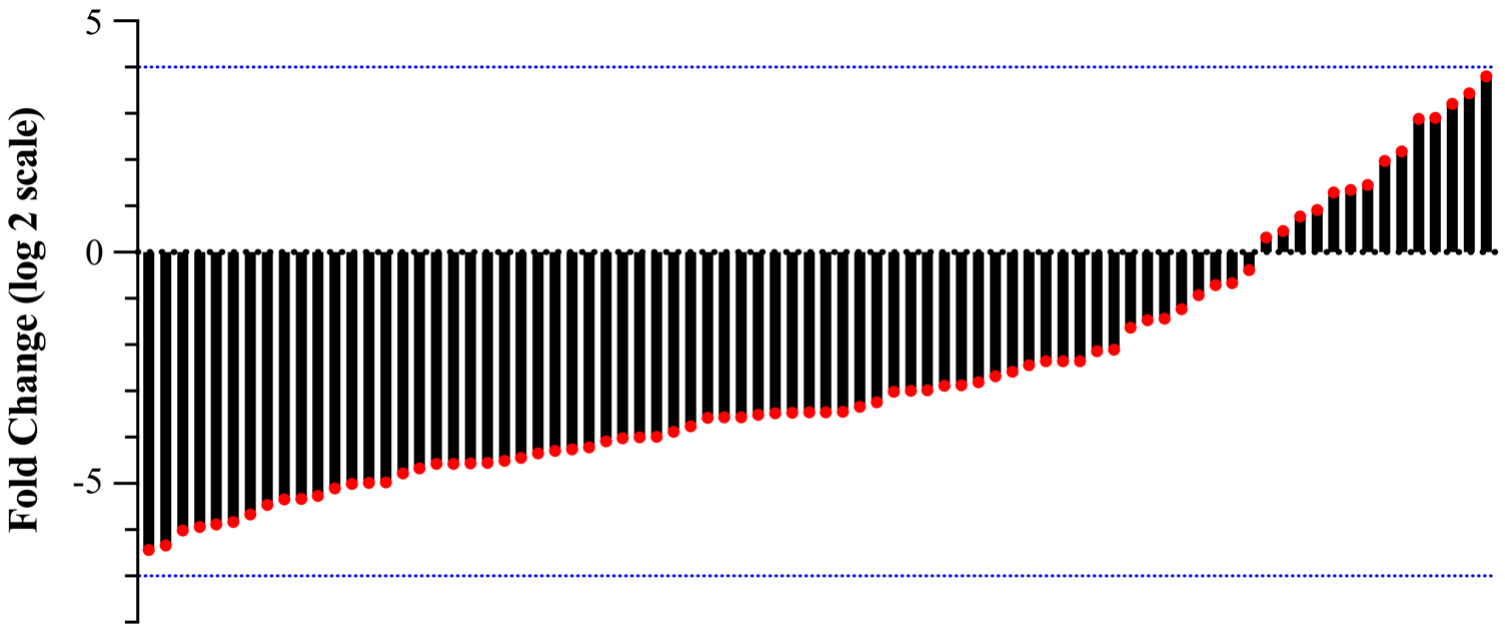
circDYRK1A was lowly expressed in GC tissues. The Y-axis is the Fold Change (log 2 scale) value. The area less than 0 means that the expression of circDYRK1A in GC tissues is lower than that in normal para-cancerous tissues, and the area more than 0 means that the expression of circDYRK1A in GC tissues is higher than that in normal para-cancerous tissues.

**Figure 10:**
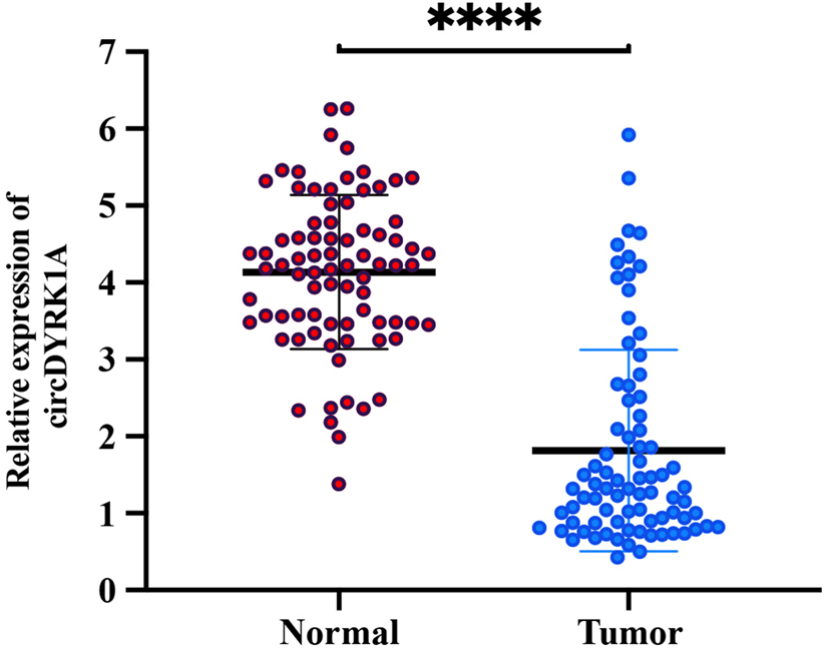
Relative expression of circDYRK1A among 80 GC tissue samples compared with adjoining healthy non-tumor counterparts. Among them, Tumor group and Normal group are the relative expression levels of circDYRK1A in tumor tissue and normal para-cancerous tissue, respectively. The Y-axis is −ΔΔCt value, and the higher the value, the higher the relative expression of circDYRK1A.

CircDYRK1A showed a decreased endogenous expression degree among GC cell lines HGC-27, AGS, and MGC-803 in comparison to that of common GES-1 while also expressed at the lowest level in MGC-803 (Fig. 8). The equivalent tendency was rather noted upon GC tissue samples in comparison to adjoining healthy counterparts, in 82.5% (66/80) of tissue samples, the expression of circDYRK1A in GC tissues was significantly lower than that in para-cancerous tissues (Fig. 9). And the relative expression of circDYRK1A in GC tissues was significantly lower than that in normal para-cancerous tissues (Fig. 10). Consequently, the association between circDYRK1A expression and significantly clinicopathological parameters with GC patients was further demonstrated that the expression patterns of circDYRK1A in corresponding tissue levels were linked with tumor invasion (*P*-value = 0.03) among GC patients (Table 1).

**Table 1:** The correlation between clinicopathological parameters with GC patients and expression of circDYRK1A.

Variables	Expression of circDYRK1A		χ^2^	*P* value
	Up	Down		
Age (y)				
≥ 60	10	36	1.347	0.246
< 60	4	30		
Gender				
Male	10	45	0.057	0.812
Female	4	21		
Tumor size (cm)				
< 3	5	17	0.574	0.449
≥ 3	9	49		
Borrmann classification				
I + II	4	12	0.779	0.377
III + IV	10	54		
Differentiation				
Well-moderate	5	23	0.004	0.951
Low	9	43		
Invasion				
T 1–2	8	18	4.698	*0.030
T 3–4	6	48		
Lymphatic metastasis				
No	5	20	0.157	0.692
Yes	9	46		
pTNM				
I–II	6	15	2.418	0.120
III–IV	8	51		

Significant values are in [bold].

Asterisk indicates a statistically significant difference (P < 0.05).

## CircDYRK1A tethers the proliferation, invasion, and migration of GC cells

**Figure 11:**
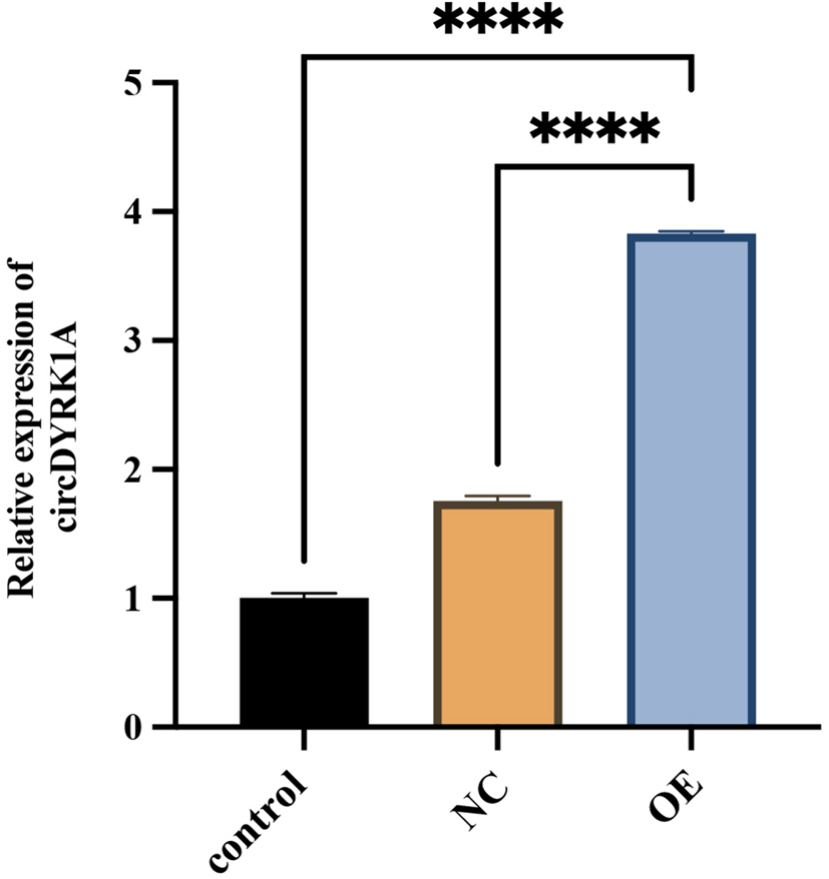
Detection of transfection efficiency of circDYRK1A in MGC-803. After transfecting by qRT-PCR, the OE (Over-Expression) group showed significantly higher expression compared with the corresponding control group and NC (Negative Control) group, indicating that the transfection was successful (*P* < 0.0001). The Y-axis is the 2^−ΔΔCt^, and the higher the value, the higher the relative expression of circDYRK1A.

**Figure 12:**
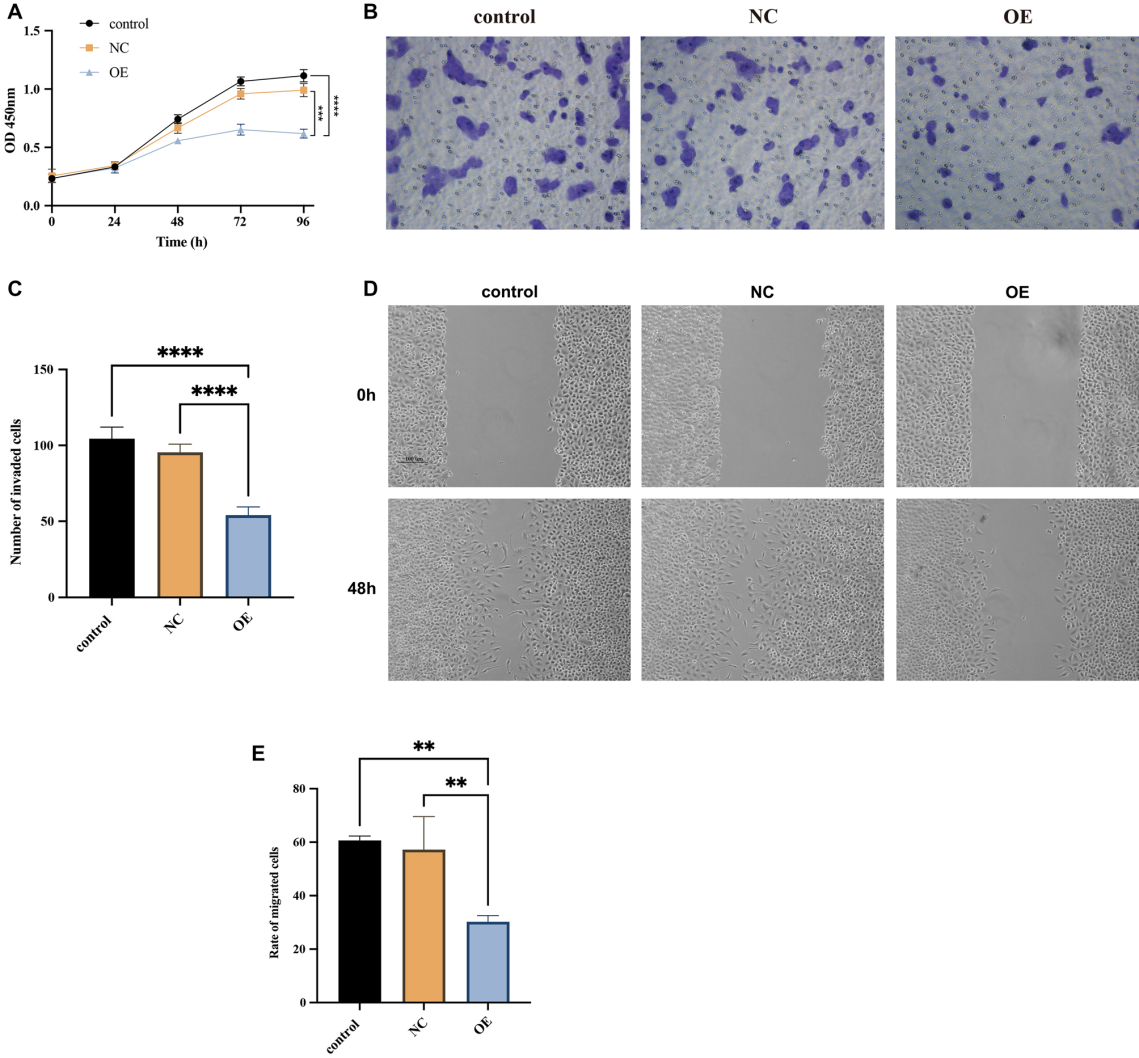
CircDYRK1A inhibited the proliferation, invasion, and migration of GC cells in vitro. (**A**) Over-expression of circDYRK1A powerfully prevented the proliferative capacity of GC cell line MGC-803. The X-axis represents the time (h) after transfection at five time points of 0, 24, 48, 72, and 96 h, respectively. The Y-axis represents the optical density (OD) value per well at a wavelength of 450 nm (OD 450). In the MGC-803 cell line, compared with the corresponding control (*P* < 0.0001) and NC (*P* < 0.001) groups, the absorbance values of the OE group were significantly lower. (**B**) Over-expression of circDYRK1A in infected MGC-803 suppressed the capability of GC cells to invade. Under the 200 × view of light microscope field, the OE group had fewer cells invaded the lower chamber of the Transwell than the corresponding control and NC groups. (**C**) Over-expression of circDYRK1A in infected MGC-803 checked the number of invaded cells. By counting the cells, the average number of the control group was 104.40 ± 7.60, the average number of the NC group was 95.40 ± 5.41, and the average number of the OE group was 54.00 ± 5.48. (**D**) Over-expression of circDYRK1A in infected MGC-803 suppressed the capability of GC cells to migrate. Under the view of inverted microscope, the healing speed of scratches in the OE group was significantly lower than that in the corresponding control and NC groups at 0 and 48 h. The scale was 100um. (**E**) Over-expression of circDYRK1A in infected MGC-803 checked the rate of migrated cells. The Y-axis represents the rate of migrated cells. By calculating the mobility, the control group is 60.64 ± 1.66, the NC group is 57.20 ± 12.41, and the OE group is 30.26 ± 2.29 (*P* < 0.01).

The over-expression molecular packing model of circDYRK1A was developed concerning its lowest presence in GC cell line MGC-803 and the overexpressed efficiency of which was verified and imagined using qRT-PCR (Fig. 11). Subsequently, CCK-8, Transwell, and Scratch Wound Healing Assays were ordinally conducted with a view to figuring out the concrete biological roles of circDYRK1A. The CCK-8 experiment concluded that up-regulated circDYRK1A was able to powerfully prevent the proliferative capacity of the selected GC cell line (Fig. 12A). In addition, the transwell experiment indicated that over-expressed circDYRK1A suppressed the capability of GC cells to invade (Fig. 12B,C). Furthermore, the scratch wound healing experiment ascertained that highly expression circDYRK1A to be a powerful inhibitor capable of restraining cell migration (Fig. 12D,E). Above all, these in vitro numerical and graphical findings totally suggested that circDYRK1A reliably deterred GC cells proliferation, invasion, and migration, specifically in cell line MGC-803.

## Discussion

Gastric cancer (GC) persists as one of the deadliest general malignancies worldwide and is a complex tumor that exhibits steadily increased incidence among less developed countries in nearly years^1^. Despite recent technical developments involving surgery and chemotherapy in antitumor strategies, worse survival in GC patients represents a considerable challenging task due to the lack of complete comprehension related to the mechanism of molecular biology studies, which limits our dominance to achieve accurate medicine. Given the prompt advances in RNA-Seq technology and bioinformatics algorithms over several decades, dysregulation of the non-coding RNAs has been identified during the occurrence of cancer initiation and progression and thus to be served as a feasible biomarker during initial diagnosis and early therapy of GC^28^. CircRNAs, a novel class of non-coding RNAs, continue to be explored in a multiplicity of diversified physiological and pathophysiological processes and have become one of the hottest focuses of tumor-related investigation^29^. In this present study, hsa_circ_0001190, also known as circDYRK1A, was ultimately determined as our research object among a series of circRNA microarray data sets according to hybrid bioinformatics algorithms. What's more, our laboratory was the first to demonstrate the expression pattern of which at the cellular and tissue levels in GC, and in parallel, their biological functions were further validated in vitro and we concluded that circDYRK1A was pinpointed to be an anti-oncogene that inhibits GC cells to proliferate, invade, and migrate. Previous literature has been reported that circDYRK1A along with several other circRNAs were down-regulated in GC, however, their analytic predictions based solely upon the side-product of a single ceRNA bioinformatics hypothesis, and the prospective evaluation and clinical implications of these modeling conclusions remain to be corroborated^30^. Unlike the former studies, circDYRK1A was eventually pinpointed to be our study subject by means of the forward (analysis from circRNA to mRNA) and reverse (retrospective analysis from mRNA to circRNA) ceRNA bioinformatics connection algorithm, at the same time, the expression pattern and the biological functions of which were supplementarily validated in vitro. More importantly, the phenomenon that the clinicopathological characteristics of GC patients manifested that downregulation of circDYRK1A was tightly correlated with increased clinical malignant progression in GC and the lower expression degree of which showed a correspondence between more advanced T stage among GC patients.

Globally, this study is not the initial exploration to contribute overview relevancy of circRNA in GC and there are numerous researches that have confirmed that incongruously expressed circRNAs were involved in the tumorigenesis and progression of GC cells. Wei et al.^31^ found that hsa_circRNA_102958 was up-regulated in GC cells and tissues, meanwhile, the expression degree of that was positively associated with the TNM stage in GC tissues and further could be a prospective biomarker for the examination of gastric carcinoma according to the significant area value under the ROC curve. Besides, Yuan et al.^32^ discovered that differentially expressed hsa_circRNA_102231 was significantly upregulated in plasma and tissue samples of GC querying GEO pubic database, which indicated to be a novel oncogene and acted as a therapeutic candidate and feasible biomarker for patients with GC. Among other malignancies, circRNA shows to take it a step further in its molecular and biological functions which were associated with carcinogenesis and development. As an example, Huang et al., discovered that hsa_circRNA_104348 was abnormally overexpressed in tissues together with cell lines of hepatocellular carcinoma (HCC), and the restraining expression of which attenuated HCC tumorigenesis and metastasis in vivo. At the same time, its potentially biological mechanism was explored that hsa_circRNA_104348 might function as a ceRNA candidate to promote HCC progression by means of targeting miR-187-3p/RTKN2 axis and initiating Wnt/β-catenin pathway^33^. More interestingly, Lin et al.^34^ further recognized that hsa_circRNA_100876 was similarly upregulated in tissues along with cells of GC and overexpression of which assisted GC cells growth, migration, and invasion. All these conclusions powerfully indicated that circRNAs were inextricably linked to the pathophysiology of cancers.

Acting as miRNA sponges to form circRNA-miRNA-mRNA triple axis is so far one of the best-illuminated functions of circRNAs in various cancer types including GC. For instance, Zhang et al.^35^ demonstrated that circNRIP1 sponged miR-149-5p thus affecting the expression degree of AKT1 and eventually promoting the progression of GC cells. Another research found that circMCTP2 was down-expressed in cisplatin-resistant GC cells along with tissues in comparison to cisplatin-sensitive GC counterparts and a low expression degree of circMCTP2 expression curbed proliferation while fostering apoptosis ability of cisplatin-resistant GC cells in responding to the remedy of cisplatin through miR-99a-5p-mediated induction of MTMR3 expression^36^.

Despite all this, however, little attention has been paid to the indicator circDYRK1A of GC. In this present research, the forward and reverse ceRNA bioinformatics connection algorithm which we creatively defined was utilized and we pinpointed that the representative hsa_circ_0001190, also known as circDYRK1A, to be chosen as our study object. At the same time, we predicted a ceRNA regulatory axis most closely related to it: circDYRK1A/miR-93-5p/TP53. Whether circDYRK1A exerts its tumor suppressor role in GC through sponging miR-93-5p, and then regulating the expression of the downstream target gene TP53? Therefore, we conducted a preliminary discussion on this regulatory axis.

As a member of the miRNA family, miR-93-5p belongs to the miR-106-25 gene cluster, and its chromosomes are all located on 7q22.1^37^. At present, a large number of literatures have reported that the abnormal expression of miR-93-5p is involved in the transition of G1/S phase of tumor cell cycle, promotes the malignant biological behavior of tumor cells, and at the same time inhibits the apoptosis function of tumor cells^38,39^. For example, Yang et al.^40^ pointed out that miR-93-5p was abnormally highly expressed in non-small cell lung cancer, and the downregulation of its expression significantly inhibited the malignant biological behavior of non-small cell lung cancer. Shen et al.^41^ analyzed differential miRNAs in GC using microarray and found that miR-93-5p was significantly highly expressed in GC tissues and multiple cell lines, and miR-93-5p could inhibit the expression of tumor suppressor gene AHNAK and promoted the epithelial-mesenchymal transition of GC, thereby promoting the occurrence and development of GC.

The TP53 gene is one of the most important genes which we identified through KEGG-related analysis, gene interaction analysis and hub gene analysis. As a common and important tumor suppressor gene, evidence shows that more than half of all malignant tumors are associated with mutations in the TP53 gene^42^. This mutation is also the most common type of genetic change in oncology research, indicating that the abnormal change of this gene is very likely to be the main pathogenic factor of tumors in organisms. In terms of its function, TP53 participates in many biological functions such as DNA damage and repair, cell cycle arrest, cell proliferation inhibition, cell apoptosis promotion, angiogenesis inhibition, so as to prevent tumorigenesis^43,44^. In GC, mutations in the TP53 gene are also very common. Busuttil et al.^45^ found that the mutation of TP53 gene was not detected in only intestinal metaplasia, but the detection rate was higher in GC, indicating that its mutation might occur in the middle and late stages of GC and played a role in the final transition stage of GC. In addition, Zha et al., found that when there was a proline genotype on codon 72 of the TP53 gene, it was closely related to the occurrence and progression of GC. Such patients not only had a poor curative effect on the chemotherapy regimen of capecitabine combined with paclitaxel, but also significantly shorten the progression-free survival and overall survival, meanwhile, related experiments also showed that its effect on the apoptosis ability of GC cells was further reduced^46^.

MiRNA can induce the degradation, deadenylation, and inhibit translation of target mRNA by complementary binding to the 3′untranslated region sequence of its target gene, thereby regulating the expression and function of the gene^47^. For the TP53 gene, many base complementary sequences can specifically bind to miRNA in its 3′ untranslated region, and miRNA can negatively regulate the activity of p53 by binding complementary to these sequences. Rahman et al.^48^ found that miR-93-5p was highly expressed in type 2 diabetes and many diseases related to the progression of the nervous system, and could interact with the TP53 gene to affect the activity of protein heterodimers and transcription factors, and then verified the interaction between miR-93-5p and TP53 gene at the theoretical level. In addition, Garbicz et al., confirmed that the expression of miR-93-5p was significantly up-regulated in invasive pituitary adenomas and could be combined with TP53 gene. miR-93-5p could serve as a new type of biomarkers to identify tumor progression and monitor postoperative tumor recurrence^49^.

Based on the above literature research and our conclusion, it is found that the mechanism of circDYRK1A regulating the expression of target gene TP53 by targeting miR-93-5p has higher credibility, and we speculate that circDYRK1A plays a role in restoring the function and activity of TP53 by sponging miR-93-5p. Taken together, these findings may be a shred of strong evidence that circDYRK1A acts as a crucial biomarker candidate to affect the biological behavior of gastric carcinoma. It regulates the expression of corresponding target genes through sponge miRNA, thereby initiating cell senescence and apoptosis, blocking cell cycle progression, and inhibiting tumor formation and development. It not only has good application prospects in the molecular therapy of malignant tumors, but also lays a new foundation for the in-depth understanding of tumor mechanisms.

## Data Availability

The datasets generated during and/or analyzed during the current study are available from the corresponding author on reasonable request.
